# Inhibition of FSTL3 abates the proliferation and metastasis of renal cell carcinoma via the GSK-3β/β-catenin signaling pathway

**DOI:** 10.18632/aging.203564

**Published:** 2021-09-23

**Authors:** Fengfeng Sun, Peng Sun, Xiaofeng Yang, Liangliang Hu, Jianguo Gao, Tao Tian

**Affiliations:** 1 Department of Urology, Zaozhuang Municipal Hospital, Zaozhuang 277100, Shandong, China

**Keywords:** FSTL3, GSK-3β, β-catenin, renal cell carcinoma, development

## Abstract

Renal cell carcinoma (RCC) is a lethal malignancy of the genitourinary system. Follistatin-like 3 (FSTL3), which mediates cell differentiation and growth, acts as a biomarker of tumors and participates in cancer development and progression. Presently, the FSTL3’s functions in RCC were investigated. Quantitative reverse transcription PCR (qRT-PCR), Western Blot, and enzyme linked immunosorbent assay (ELISA) were conducted to verify FSTL3 expression in RCC tissues and cell lines. BrdU assay and CCK8 experiment were made to monitor cell proliferation. Transwell was implemented to examine the invasion of the cells. Flow cytometry analyzed cell apoptosis, and Western Blot evaluated the protein levels of E-cadherin, Twist, and Slug. In the meantime, the protein profiles of the GSK-3β, β-catenin, and TGF-β signaling pathways were ascertained. Moreover, the Xenograft tumor model was constructed in nude mice for measuring tumor growth *in vivo*. The statistics showed that FSTL3 presented an overexpression in RCC, and patients with a lower expression of FSTL3 manifested a better prognosis. Down-regulated FSTL3 hampered the proliferation, invasion, EMT, and tumor growth of RCC cells and caused cell apoptosis. On the contrary, FSTL3 overexpression enhanced the malignant behaviors of RCC cells. Furthermore, FSTL3 knockdown bolstered GSK-3β, suppressed β-catenin, and reduced BMP1-SMAD pathway activation. Inhibited β-catenin substantially mitigated FSTL3-mediated promoting functions in RCC. In short, FSTL3 functions as an oncogene in RCC by modulating the GSK-3β/β-catenin signaling pathway.

## INTRODUCTION

Renal cell carcinoma (RCC) has its roots in epithelial cells of the renal parenchyma. RCC is the third most malignant tumor following prostate cancer and bladder cancer. In light of statistics, the incidence of RCC has been on the increase year by year, and the mortality rate is over 40% [1]. In the early stage, RCC is applicable to surgical resection, but one-third of RCC patients exhibit metastases at the time of definite diagnosis. These patients have a high surgical recurrence rate, poor response to chemoradiotherapy, and low response rate to immunotherapy [2]. Therefore, for advanced treatment strategies, a deeper understanding of RCC's pathogenesis and new biomarkers are warranted.

Glycogen synthase kinase-3β (GSK-3β) is a serine/threonine kinase extensively implicated in cell cycle modulation, apoptosis, DNA repair, and resistance of chemotherapy and radiotherapy in the pathogenesis of multiple cancers [3–4]. For instance, GSK-3β overexpression boosts breast cancer cell migration and dampens autophagy activation by modulating the AMPK pathway [5]. On the other hand, β-catenin (encoded by CTNNB1), a subunit of the cell surface cadherin protein compound serving as an intracellular signal transducer in the WNT signaling pathway, can also interact with the other transcription factors, covering T-cell factor, forkhead box protein O, and hypoxia inducible factor 1α, to modulate the profiles of target genes [6]. Therefore, β-catenin facilitates the transcription of various oncogenes, covering c-Myc and CyclinD-1, leading up to carcinogenesis and tumor progression of several cancers [7]. Interestingly, more and more studies imply that the GSK-3β/β-catenin pathway exerts a function in tumor development [8]. For example, in epithelial ovarian cancer, Emodin (EMO) represses the invasion, migration, and epithelial mesenchymal transition (EMT). Additionally, EMO abates glycogen synthase kinase 3β (GSK-3β) phosphorylation, attenuates the level of total β-catenin protein, and down-regulates transcription factor zinc finger E-box binding homeobox 1 (ZEB1) [9]. What’s more, miR-155 [10] and miR-1246 [11] both function as oncogenes in cancers via directly targeting the GSK-3β-mediated Wnt/β-Catenin pathway. Thus, GSK-3β/β-catenin pathway regulation may be a prospective method for RCC treatment.

Follistatin-like 3 (FSTL3), also called follistatin-related protein or follistatin-related gene (FLRG) protein, is a highly conserved monomer secreted glycoprotein located on chromosome 19p13.3. *FSTL3,* about 7 kb in length, is comprised of five exons and four introns, which encode a signal peptide, an N-terminal domain, two FS regions, and a C-terminal domain [12]. Via its combination with the members of the transforming growth factor beta (TGFβ) superfamily, such as activin A and myostatin, FSTL3 takes part in the modulation of diverse biological effects [13]. Recent literatures have revealed that FSTL3 mediates metabolic homeostasis [14], myocardial ischemic injury [15], systemic sclerosis-correlated pulmonary hypertension [16], and other essential physiological and pathological mechanisms. Behnke et al. have discovered an overexpression of FSTL3 in 100% HCC samples, signifying a diagnostic biomarker [17]. Gao et al. have also disclosed that lncRNA DSCAM AS1 targeting miR-122-5p uplifts FSTL3 expression, hence bolstering non-small cell lung cancer proliferation, migration, and invasion [18]. Unfortunately, FSTL3 is barely investigated in kidney diseases, encompassing RCC.

Existing papers have identified that TGF-β/Smad signal activation is intricately associated with RCC occurrence and growth [19]. Interestingly, Liu YJ et al. have uncovered that FSTL3 is up-regulated in gastric cancer and activates epithelial-mesenchymal transition (EMT) by enhancing F-actin profile and BMP/SMAD signaling [20]. Therefore, we conjectured that FSTL3 influences RCC progression by modulating the TGF-β/Smad signal. Our discoveries validated that FSTL3 expression was augmented in RCC. FSTL3 up-regulation boosted β-catenin and TGF-β/Smad signal activation. Thus, we performed further experiments to figure out the functions of FSLT3 in RCC, with an eye to offering new insights into RCC treatment.

## RESULTS

### FSTL3 expression in RCC tissues and cells

**Figure 1:**
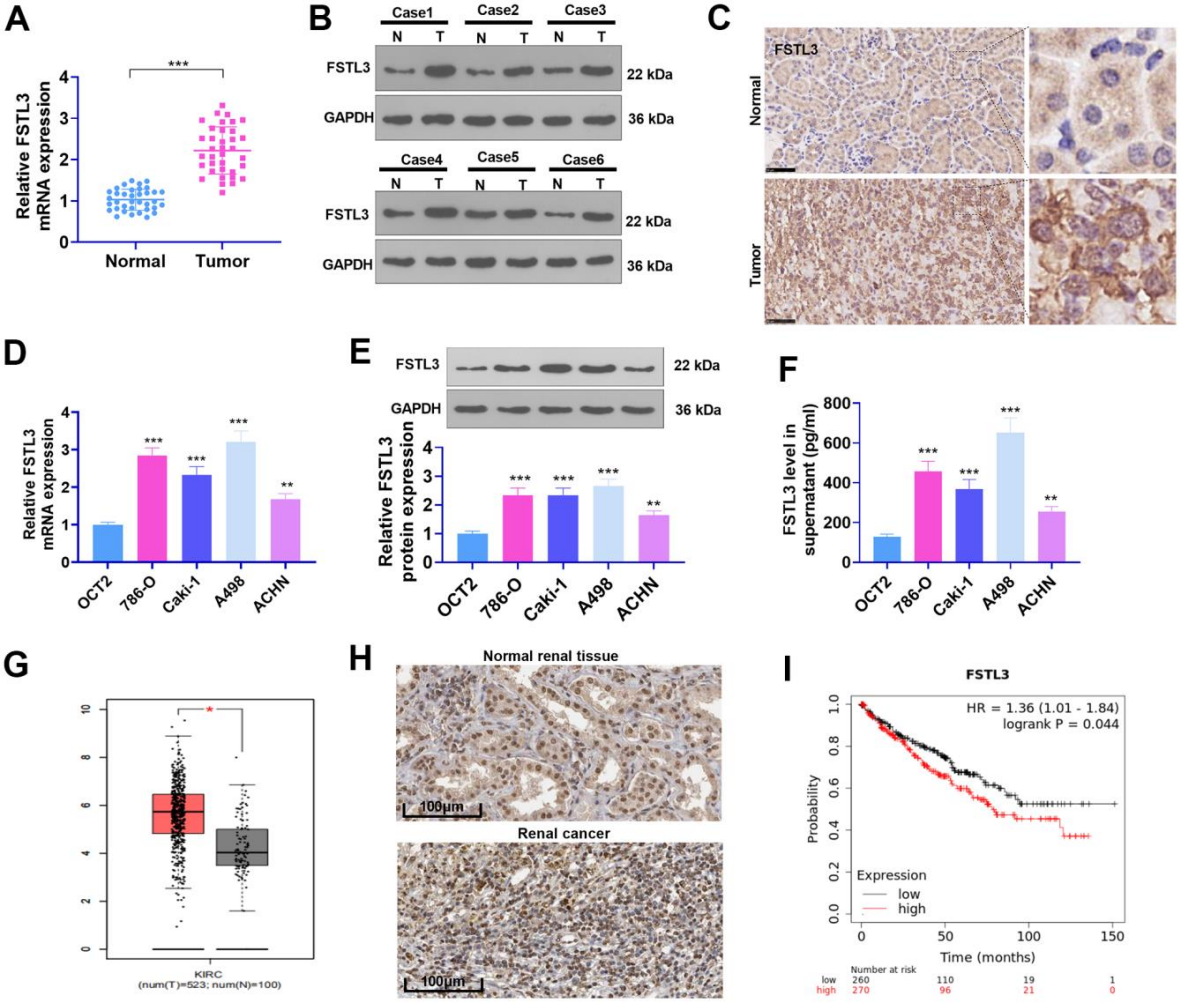
**The profile of FSTL3 in RCC.** (**A**) qRT-PCR determined FSTL3’s expression in RCC tissues as well as adjacent normal tissues, ***P<0.001 (vs. Normal group); (**B**) Western blot was taken for FSTL3 profile detection in RCC tissues and adjacent normal tissues. (**C**) IHC ascertained FSTL3’s expression in RCC tissues and adjacent normal tissues, Bar=50 μm; (**D**, **E**) qRT-PCR (**D**) and Western Blot (**E**) examined FSTL3 in the OCT2, A498, 786-O, Caki-1, and ACHN cell lines, **P<0.01, ***P<0.001 (vs. the OCT2 group). N=5. (**F**) ELISA measured FSTL3 in the OCT2, A498, 786-O, Caki-1, and ACHN culture mediums, **P<0.01, ***P<0.001 (vs. the OCT2 group). (**G**) FSTL3 expression in RCC was figured out through the GEPIA database (http://gepia.cancer-pku.cn/). (**H**) FSTL3’s expression in RCC tissues or normal renal tissues was analyzed via Human Protein Atlas (https://www.proteinatlas.org/). (**I**) The Kaplan-Meier Plotter database (http://kmplot.com/analysis/index.php?p) was consulted to confirm FSTL3 expression in the survival rate of KIRC patients.

qRT-PCR, western blot, and IHC determined FSTL3 expression in RCC tissues and the compared non-tumor tissues. The result demonstrated that the mRNA and protein levels of FSTL3 were highly expressed in RCC tissues vis-a-vis adjacent normal tissues (*P*<0.05, Figure 1A–1C). Moreover, in contrast with the normal human proximal tubular epithelial cell line (OCT2), FSTL3 profile in RCC cell lines (786- O, Caki-1, A498, ACHN) were considerably elevated (*P*<0.05, Figure 1D–1F). Then we browsed GEPIA (http://gepia.cancer-pku.cn/). The biological information analysis revealed that FSTL3 expression in clear renal cell carcinoma (KIRC) was notably higher than that in normal tissues adjoining cancer (Figure 1G). Through the Human Protein Atlas (https://www.proteinatlas.org/), FSTL3 was discovered to be moderately expressed in normal renal tissues but strongly expressed in RCC tissues (Figure 1H). The Kaplan-Meier Plotter (http://kmplot.com/analysis/index.php?p) disclosed that the survival rate of those with high FSTL3 profile was much lower than those with low FSTL3 profile (Figure 1I). These discoveries signified that FSTL3 exhibited an overexpression in RCC and might be a novel biomarker for RCC in forecasting the survival rate of RCC patients.

### FSTL3 overexpression facilitated RCC proliferation and invasion and repressed cell apoptosis

**Figure 2:**
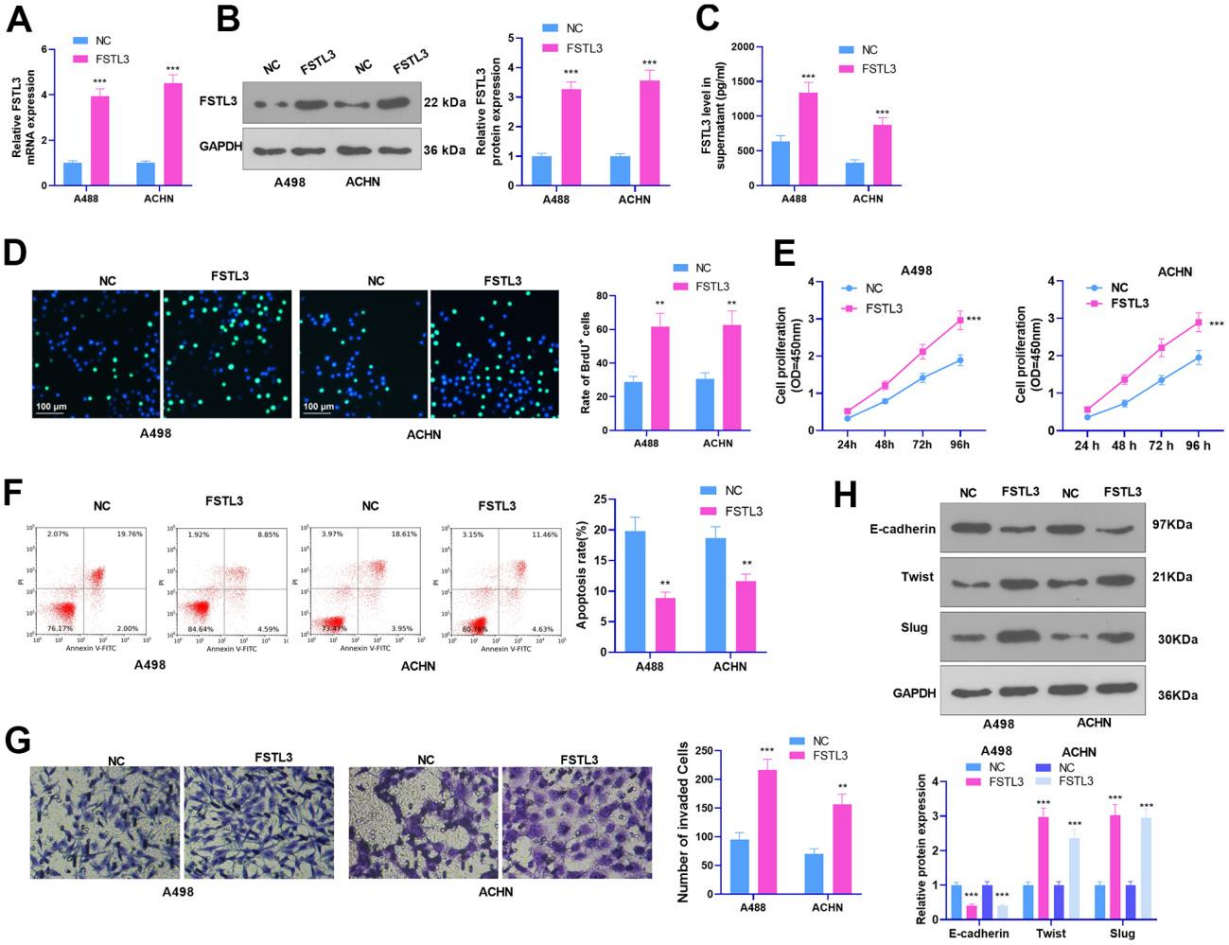
**FSTL3 overexpression facilitated RCC proliferation and metastasis and cramped cell apoptosis.** A498 and ACHN cells were transiently transfected along with FSTL3 overexpression plasmids. (**A**–**C**) qRT-PCR (**A**), Western Blot (**B**), and ELISA (**C**) were done for FSTL3 detection; (**D**, **E**) BrdU (**D**) and CCK8 (**E**) were implemented to examine cell proliferation; (**F**) Flow cytometry measured cell apoptosis; (**G**) Transwell evaluated cell invasion; (**H**) Western Blot assessed E-cadherin, Twist, and Slug expression in RCC cells. **P<0.01, ***P<0.001 (vs. the NC group). N=5.

To probe into the function of FSTL3 in RCC progression, we set up an FSTL3 overexpression model in the A498 and ACHN cell lines. As FSTL3 was remarkably up-regulated in the two cells, FSTL3 release in the culture medium was enhanced (*P* < 0.05, Figure 2A–2C). BrdU assay tested cell proliferation. It reflected that FSTL3 overexpression stimulated increased BrdU-positive cell rate (*P* < 0.05, Figure 2D). The results of CCK8 were found akin to those of BrdU. Following FSTL3 overexpression, the cell proliferation was greatly expanded (*P* < 0.05, Figure 2E). Flow cytometry unraveled that the apoptosis rate of overexpressed FSTL3 cells declined substantially, lower than that of the NC group (*P* < 0.05, Figure 2F). Transwell manifested that the invasion of cells post FSTL3 overexpression was prominently strengthened (*P* < 0.05, Figure 2G). Besides, Western Blot for epithelial-mesenchymal transformation (EMT) markers (E-cadherin, Twist, and Slug) unveiled that FSTL3 overexpression heightened the levels of Twist and Slug but curbed E-cadherin expression (*P* < 0.05, Figure 2H, in contrast with the NC group). These findings denoted that FSTL3 boosted the proliferation, invasion, and EMT of RCC cells and cramped RCC apoptosis.

### FSTL3 knockdown hampered the malignant phenotypes of RCC

**Figure 3:**
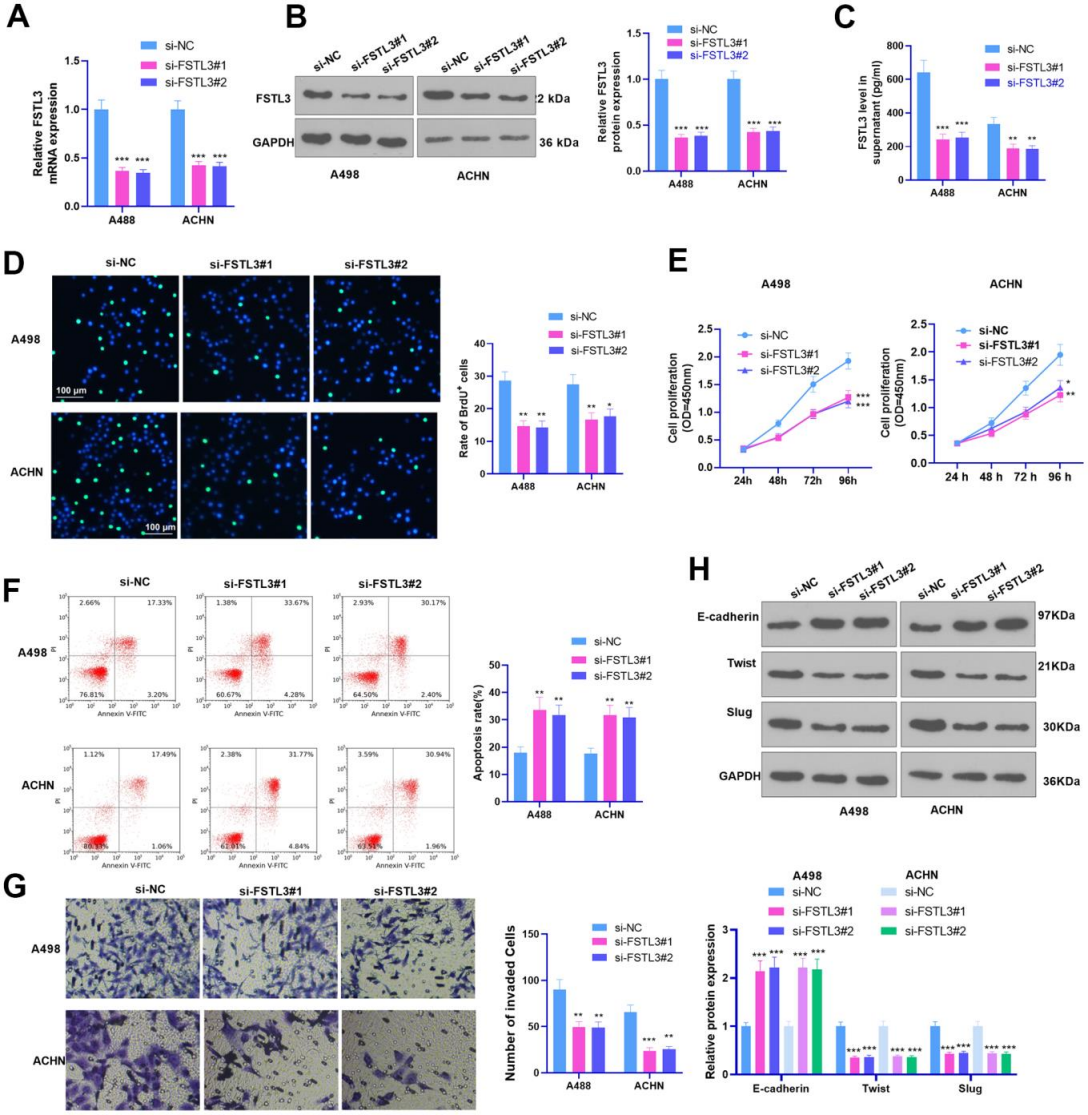
**FSTL3 knockdown impeded RCC proliferation and metastasis and accelerated cell apoptosis.** A498 and ACHN cells were transiently transfected along with si-FSTL3. (**A**–**C**) qRT-PCR (**A**), Western Blot (**B**), and ELISA (**C**) were taken for FSTL3 detection; (**D**, **E**) BrdU (**D**) and CCK8 (**E**) analyzed cell proliferation; (**F**) Flow cytometry examined cell apoptosis; (**G**) Transwell assessed cell invasion; (**H**) Western Blot evaluated E-cadherin, Twist, and Slug expression in RCC cells. *P<0.05, **P<0.01, ***P<0.001 (vs. the si-NC group). N=5.

A low FSTL3 expression model was established in the A498 and ACHN cell lines. The results of qRT-PCR, Western Blot, and ELISA identified that FSLT3 was down-regulated in the si-FSLT3 group (against the si-NC group, Figure 3A–3C). BrdU assay, CCK8 assay, flow cytometry, Transwell, and Western Blot examined cell proliferation, apoptosis, invasion, and EMT, respectively. The experimental outcomes illustrated that FSTL3 knockdown notably suppressed BrdU-positive cell rate, proliferation, and invasion and augmented cell apoptosis (*P* < 0.05, Figure 3D–3G). EMT protein expression was ascertained by Western Blot. It was validated that E-cadherin expression was considerably higher than the si-NC group after FSTL3 knockdown, whereas the protein profiles of Twist and Slug were lowered (*P* < 0.05, Figure 3H). The above outcomes implied that FSTL3 knockdown dampened RCC’s malignant behaviors and cell EMT.

### FSTL3 release inhibition repressed RCC proliferation and invasion and enhanced cell apoptosis

**Figure 4:**
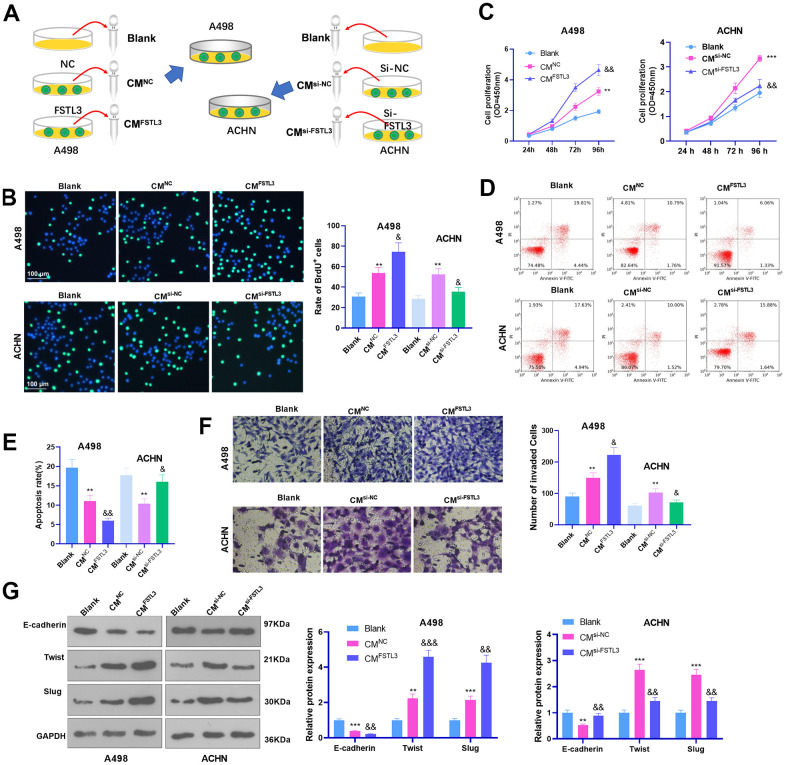
**FSTL3 knockdown suppressed RCC proliferation and metastasis and boosted cell apoptosis.** (**A**) The conditioned medium (CM) of RCC cells with FSLT3 overexpression or down-regulation was collected. RCC cells were with the CM. A498 and ACHN cells were transiently transfected along with si-FSTL3. (**B**, **C**) BrdU (**B**) and CCK8 (**C**) monitored cell proliferation. (**D**, **E**) Flow cytometry examined cell apoptosis; (**F**) Transwell assessed cell invasion; (**G**) Western Blot evaluated E-cadherin, Twist, and Slug profile in RCC cells. *P<0.05, **P<0.01, ***P<0.001 (vs. the Blank group). &*P*<0.05, &&*P*<0.01, &&&*P*<0.001 (vs. the CM^NC^ or CM^si-NC^ group). N=5.

Since FSLT3 is a secreted protein, we further collected the conditioned medium (CM) of RCC cells with FSLT3 overexpression or down-regulation and treated RCC cells with the CM (Figure 4A). Cell proliferation was examined via BrdU and CCK8. It was discovered that by contrast to the blank group, both CM^NC^ and CM^si-NC^ enhanced cell proliferation (Figure 4B, 4C). Enhanced FSTL3 bolstered RCC cell proliferation, while FSTL3 knockdown attenuated the proliferation (in contrast with CM^NC^ or CM^si-NC^, Figure 4B, 4C). Flow cytometry revealed that the apoptosis rate in CM^NC^ or CM^si-NC^ was abated vis-a-vis the blank group (Figure 4D, 4E). The CM from FSTL3-overexpressed cells vigorously decreased cell apoptosis (by contrast to the CM^NC^ group), while the CM from FSTL3-downregulated cells substantially enhanced cell apoptosis (against the CM^si-NC^ group, Figure 4D, 4E). Transwell and Western Blot confirmed that the invasive ability and EMT of RCC cells were stepped up by CM^NC^ or CM^si-NC^. FSTL3 overexpression strengthened that effect (compared with CM^NC^ group), while FSTL3 knockdown inverted that effect (in contrast with the CM^si-NC^ group, Figure 4F, 4G). Taken together, secreted FSTL3 boosted RCC cell proliferation, invasion, and EMT and hampered RCC apoptosis.

### FSTL3 up-regulation stepped up RCC cell growth *in vivo*

**Figure 5:**
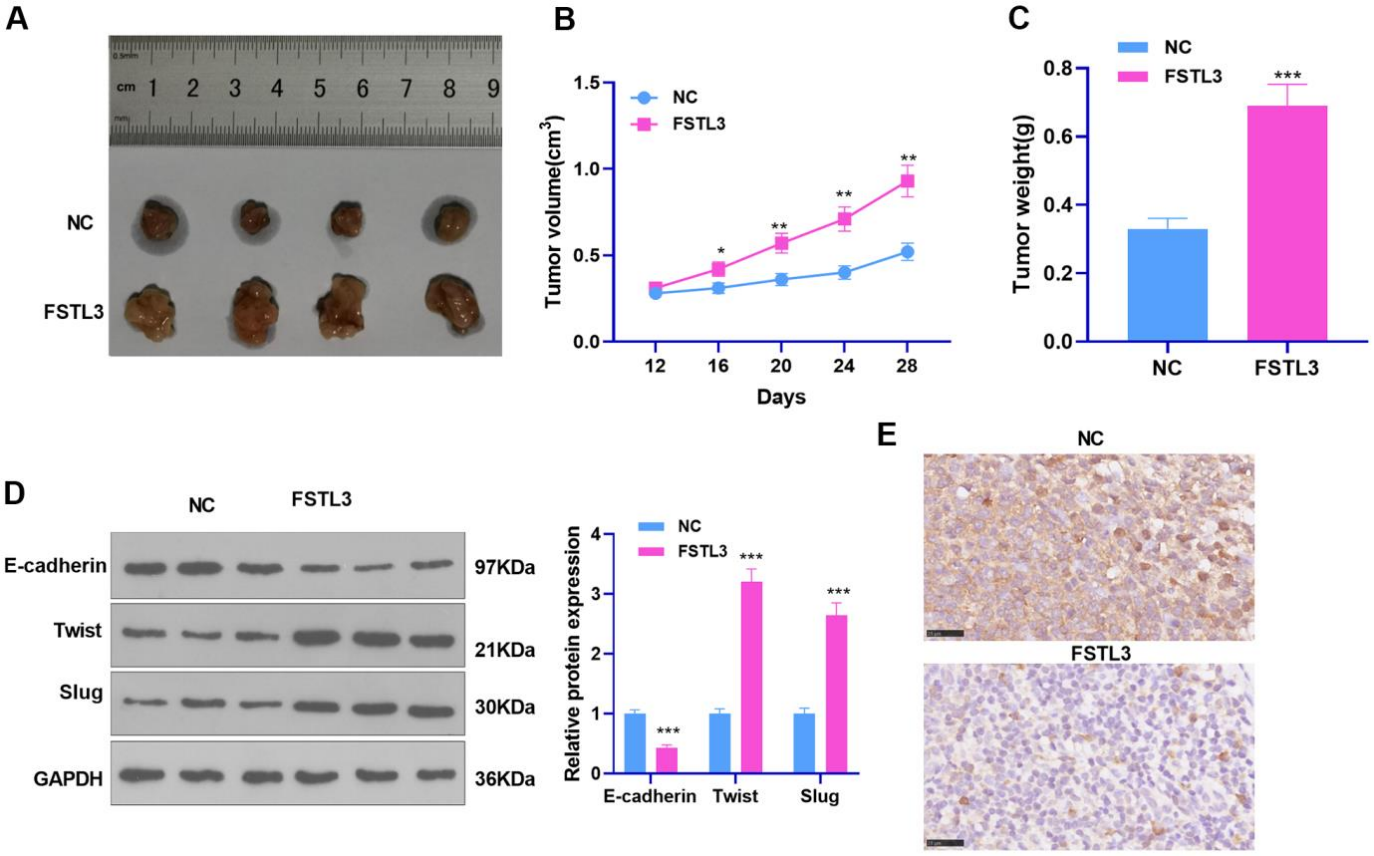
**FSTL3 influenced RCC growth.** ACHN cells transiently transfected along with the overexpression plasmids of FSTL3 were transfused under the skin of the experimental mice. (**A**–**C**) We observed and measured the tumor growth in mice and drew the growth curve and weighted histogram; (**D**) Western Blot evaluated the protein profiles of E-cadherin, Twist, and Slug. (**E**) IHC determined E-cadherin expression in the tumor tissues. *P<0.05, **P<0.01, ***P<0.001 (vs. the NC group). N=5.

The influence of FSTL3 on RCC was further researched *in vivo*. The tumor formation model in nude mice was erected through the subcutaneous injection of A498 cells transiently transfected with FSTL3-overexpressed plasmids, with the tumor growth in mice observed and recorded. It was discovered that FSTL3 enhanced tumor growth in mice, which manifested that the tumor volume and mass were larger than the NC group (*P* < 0.05, Figure 5A–5C). EMT protein expression in tissues determined by Western Blot indicated that Twist and Slug expression in the FSTL3 group were heightened, whereas E-cadherin expression was abated (*P* < 0.05, Figure 5D, in contrast with the NC group). Furthermore, IHC was implemented to examine E-cadherin in the formed tumor tissues. It was unraveled that by contrast to the NC group, E-cadherin expression in the tumor cells was remarkably uplifted (Figure 5E). This phenomenon denoted that FSTL3 not only promoted RCC growth *in vivo* but also incurred EMT.

### FSTL3 modulated GSK-3β/β-catenin and BMP1/SMAD pathway activation *in vitro* and *in vivo*

**Figure 6:**
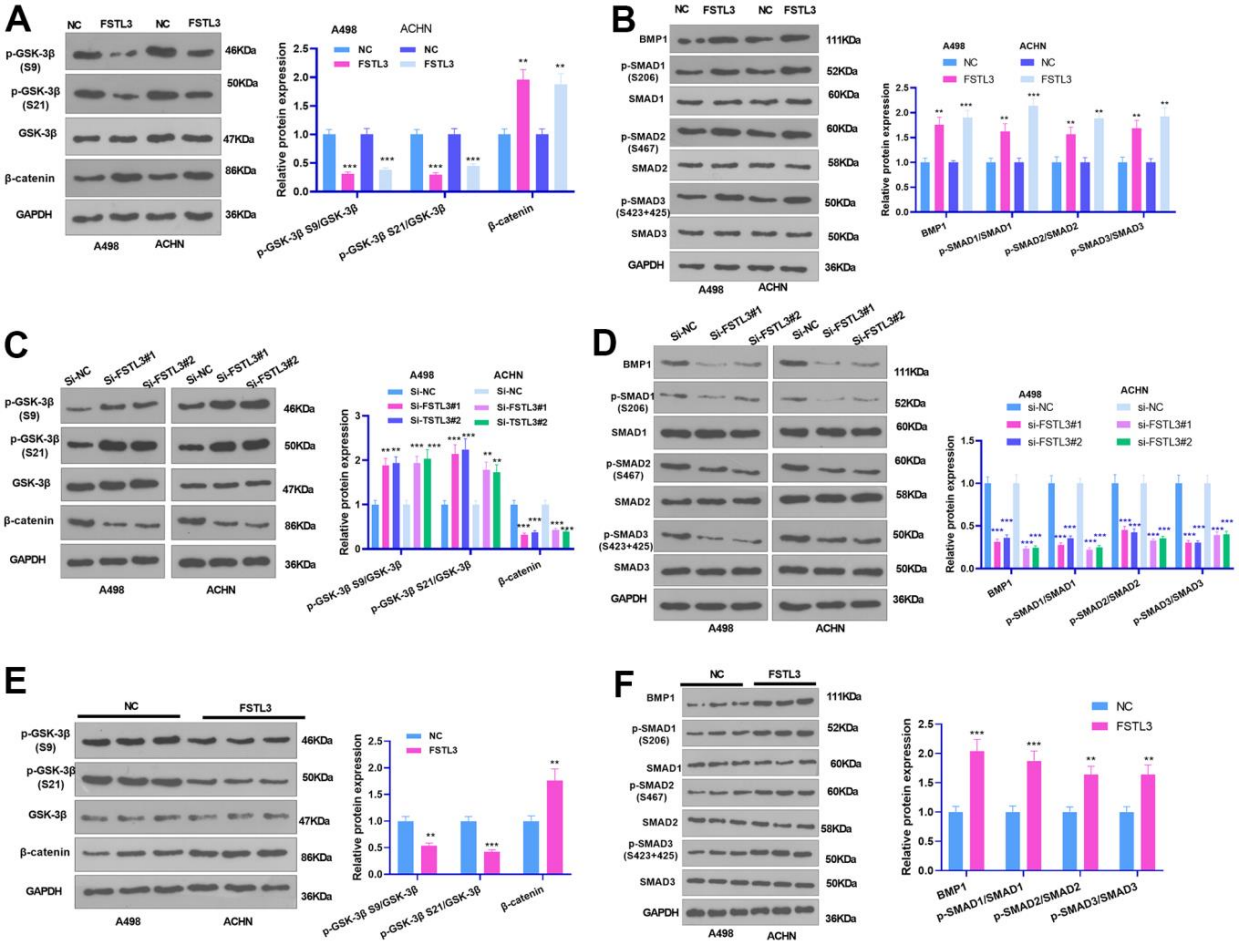
**FSTL3 modulated GSK-3β/β-catenin pathway activation.** (**A**, **B**) The A498 and ACHN cells were transiently transfected along with the overexpression plasmids of FSTL3. Western Blot experiment was done to verify the protein expressions of GSK-3β/β-catenin, and BMP1, SMAD1, SMAD2, and SMAD3 in RCC cells. (**C**, **D**) The A498 and ACHN cells were transiently transfected along with si-FSTL3. Western Blot was implemented to check the protein profiles of GSK-3β/β-catenin, and BMP1, SMAD1, SMAD2, and SMAD3 in RCC cells. (**E**, **F**) Western blot examined GSK-3β, β-catenin, BMP1, SMAD1, SMAD2, and SMAD3 **i**n the formed tumor tissues. **P<0.01, ***P<0.001 (vs. NC or si-NC group). N=5.

To clarify the mechanism by which FSTL3 plays a pro-oncogenic role in RCC, we further measured the profiles of the GSK-3β/β-catenin and BMP1/SMAD signals after up-regulating or down-regulating FSTL3. The results illustrated that FSTL3 overexpression repressed GSK3 beta (phospho S9) and GSK3 alpha (phospho S21) and notably brought up the levels of β-catenin, BMP1, p-SMAD1 (S206), p-SMAD2 (S467), and p-SMAD3 (S423+S425) (Figure 6A, 6B). In contrast, FSTL3 knockdown restricted the levels of β-catenin, BMP1, p-SMAD1 (S206), p-SMAD2 (S467), and p-SMAD3 (S423+S425) but augmented the profiles of GSK3 beta (phospho S9), and GSK3 alpha (phospho S21) (Figure 6C, 6D). GSK3 beta (phospho S9) and GSK3 alpha (phospho S21) were attenuated, and the levels of β-catenin, BMP1, p-SMAD1 (S206), p-SMAD2 (S467), and p-SMAD3 (S423+S425) were elevated in the formed tumor tissues following FSTL3 overexpression (P < 0.05, Figure 6E, 6F). Those discoveries reflected that FSTL3 overexpression activated the β-catenin and BMP1-SMAD signaling pathways.

### β-catenin inhibition alleviated FSTL3-triggered oncogenic effects

**Figure 7:**
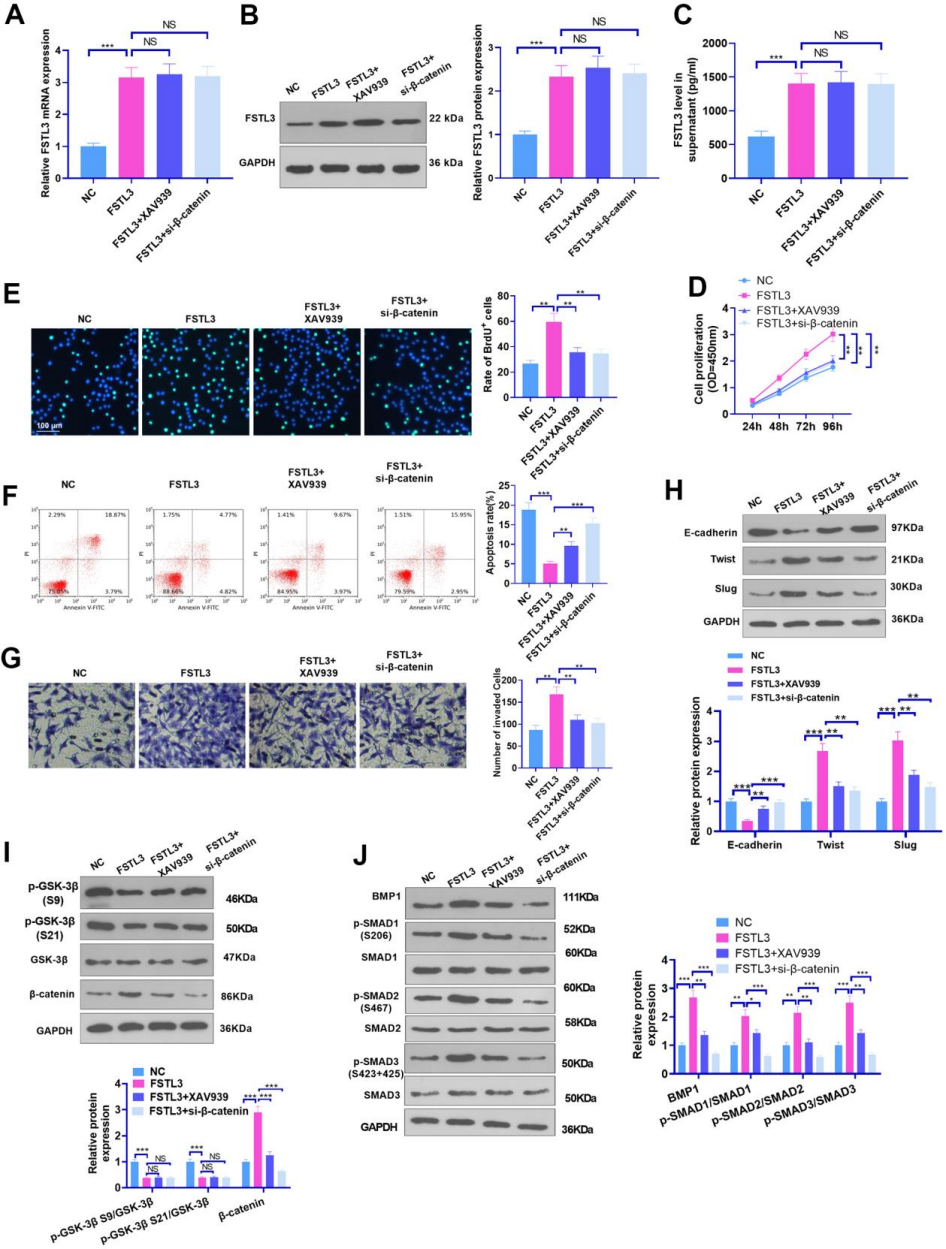
**β-catenin inhibition attenuated FSTL3-induced RCC progression.** FSTL3 overexpression plasmids were transfected into ACHN cells, and XAV939 (1 μM) or si-β-catenin was added for intervention post FSTL3 overexpression. (**A**–**C**) qRT-PCR (**A**), western blot (**B**), and ELISA (**C**) were deployed for FSTL3 detection; (**D**–**G**) CCK8 (**D**), BrdU (**E**), Flow cytometry (**F**), and Transwell (**G**) monitored cell proliferation, apoptosis, and invasion; (**H**–**J**) The protein expressions of the EMT, GSK-3β/β-catenin, and BMP1/SMAD pathways were confirmed via Western Blot. *P<0.05, **P<0.01, ***P<0.001. N=5.

To grasp whether FSTL3 inhibition would alter the occurrence and progression of RCC, we suppressed β-catenin using its inhibitor XAV939 (1 μM) or si-β-catenin based on FSTL3 overexpression in ACHN cells. qRT-PCR, western blot, and ELISA examined FSTL3 expression. It turned out that FSTL3 expression in the FSTL3+XAV939 and FSTL3+si-β-catenin groups was not considerably altered in contrast with the FSTL3 group (P > 0.05, Figure 7A–7C). BrdU, CCK8, flow cytometry, and Transwell validated that compared to the FSTL3 group, the cell proliferation and invasion were vigorously mitigated after XAV939 treatment or si-β-catenin transfection, while the apoptosis was stepped up (P < 0.05, Figure 7D–7G). Then, the protein profiles of the EMT and GSK-3β/β-catenin pathways, and the BMP1/SMAD pathway were ascertained via Western Blot. The statistics implied that XAV939 and si-β-catenin reversed FSTL3-incurred EMT, β-catenin, and BMP1/SMAD pathway up-regulation (P < 0.05), coupled with no largely altered levels of GSK3 beta (phospho S9) and GSK3 alpha (phospho S21) (P > 0.05, Figure 7H–7J). Therefore, β-catenin inhibition attenuated the oncogenic function triggered by FSTL3.

## DISCUSSION

RCC, the most prevalent renal malignancy, makes up 3% of malignancies in adults [21]. RCC incidence is on the rise year by year. Owing to the lack of typical clinical manifestations and certain diagnostic markers, some patients have reached the advanced stage of the disease when they are clearly diagnosed. RCC exhibits a poor prognosis with a five-year survival rate of only about 10%, especially for patients displaying metastasis [22]. Notwithstanding, the treatment options available to patients with metastatic RCC are limited. This study has initially discovered that FSTL3, which is up-regulated in RCC, boosts RCC progression via the GSK-3/β-catenin pathway, creating a new effective impetus for RCC patients' prognosis improvement.

As secreted follistatin-module-containing glycoproteins, several FSLT members, covering FSTL1 [23, 24] and FSTL5 [25, 26], have been discovered to participate in cancer cell growth, stemness, chemoresistance, invasion, and migration. As for FSTL3, it has been demonstrated to mediate various biological processes. For instance, Xia et al. have suggested that FSTL3 overexpression influences gonadal development and function. Subsequent studies have progressively corroborated that FSTL3 makes a great contribution to cancer development [27]. Panagiotou et al. have confirmed that higher FSTL3 expression positively correlated with malignant breast cancer boasts the potential to be a diagnostic biomarker [28]. Zawadzka et al. have manifested that FSTL3 may be a candidate biomarker for breast cancer [29]. Moreover, Gao et al. have pinpointed that FSTL3 is a brand new oncogene of non-small cell lung cancer (NSCLC), and FSTL3 inhibition impedes NSCLC development, migration, and invasion [18]. At present, it has been revealed that FSTL3 has an enhanced expression in A498, 786-O, Caki-1, and ACHN RCC cells by contrast to that in OCT2 cells (a normal human proximal renal tubular epithelial cell line). Furthermore, it transpires that FSTL3 presents a higher level in RCC tissues than that in adjacent normal tissues. Here, all the A498 [30], 786-O [31], Caki-1 [32], and ACHN [33] cells were adopted for both *in-vitro* and *in-vivo* experiments to interrogate the mechanism of RCC progression. The FSTL3 overexpression and knockdown models were both set up in A498 and ACHN cells. The findings reflected that FSTL3 not only boosted RCC proliferation, invasion, EMT, and growth, but also dampened cell apoptosis. Hence, FSTL3 is a promising diagnostic and therapeutic target in RCC.

Glycogen synthase kinase-3β (GSK-3β) has been discovered to impact cell growth [34], metastasis [35], immune response [36], angiogenesis [37], and neural development [38]. Nonetheless, dual roles of GSK-3β in tumor development have been revealed to incur apoptosis and enhance proliferation in multiple diseases. As a result, GSK-3*β* may serve as a related complementary strategy for antibiotic treatment, forging an interesting scenario in the development of new antimicrobial strategies [39]. In renal cancer, prior studies have signified that the pharmacological inhibition of GSK-3 by SB-216763, TDZD8, or 9-ING-41 represses renal cancer cell growth [40, 41]. Nevertheless, GSK-3β protein level increased either by the chemical agent such as 6-Gingerol [42] or its upstream molecular lncRNA NBAT1/miR-346 [43] can hinder cell proliferation and migration in renal cancer. Interestingly, a preceding study has uncovered that FSTL1 aggravates hepatocellular carcinoma progression via inducing AKT/GSK-3β pathway activation [44]. Here, we figured out that GSK3 beta (phospho S9) and GSK3 alpha (phospho S21) were both down-regulated post FSTL3 up-regulation, and their levels were brought up in FSTL3-downregulated RCC cells. The different downstream proteins modulated by GSK-3β might be implicated in this phenomenon.

The Wnt signaling pathway has been uncovered to exert critical functions in tumorigenesis by modulating cell proliferation, apoptosis, differentiation, polarization, and migration [45]. In RCC, Wnt signaling pathway activation partakes in cancer metastasis via enhancing migration, invasion, and EMT [46, 47]. Following Wnt pathway activation, β-catenin was accumulated in the cytoplasm and translocated into the nucleus. As a consequence, it is combined to LEF-1/TCF4 and some other co-regulators to step up the transcription of target genes, covering Jun, c-Myc, and CyclinD-1, in a tissue-specific pattern, most of which encode oncoproteins [7]. Moreover, the Wnt/β-catenin pathway curbs E-cadherin expression [48] and promotes SLUG [49] and Twist [50]. These works have confirmed that the Wnt/β-catenin pathway exerts a vital function in mediating EMT. Interestingly, Kelaini et al. have revealed that FSTL3 can trigger and enhance endothelial features by facilitating β-catenin nuclear translocation via glycogen synthase kinase-3β activity inhibition [51]. This paper displayed that FSTL3 exhibited a potential function in modulating the GSK-3β/β-catenin pathway. Similarly, FSTL1 steps up chemoresistance and sustains stemness in breast cancer cells via the integrin β3/Wnt/β-catenin pathway [23]. Here, it was uncovered that β-catenin level was markedly heightened subsequent to FSTL3 overexpression. Yet, β-catenin inhibition contributed to mitigated malignant behaviors of RCC cells. Hence, we believed that FSLT3 boosted RCC development dependently via the GSK-3β/β-catenin pathway.

As a pivotal mediator in tumor development, transforming growth factor-β1 (TGF-β1) is embroiled in mediating carcinogenesis and metastasis through activating its downstream small mother against decapentaplegic (Smad) signaling [52, 53]. Recent works have revealed that Wnt/β-catenin pathway inhibition inverts TGF-β-mediated EMT of tumor cells [54, 55]. Growth differentiation factor 8 (GDF8) facilitates cell invasiveness in human trophoblasts by uplifting FSTL3 expression through the ALK5-SMAD2/3 signaling pathway [56]. FSTL3 bolsters the BMP/SMAD pathway [20]. By implementing *in-vitro* and *in-vivo* experiments, we ascertained that FSTL3 overexpression enhanced BMP1 and SMAD1/2/3 phosphorylation. β-catenin inhibition weakens the BMP1/SMAD pathway, indicating that FSTL3 potentially exerts an oncogenic function via inducing the TGF-β and Wnt/β-catenin pathways.

To summarize, FSTL3 overexpressed in RCC boosts RCC development and growth by inducing the TGF-β and Wnt/β-catenin pathways. Our research creates a new impetus for the development of novel RCC therapies and the improvement of patient prognosis. Notwithstanding, this paper lacks clinical case data, and experimental animals are relatively limited. Therefore, further analysis and research are required in combination with the clinical characteristics of RCC patients to facilitate RCC treatment development.

## MATERIALS AND METHODS

### Cell culture and treatment

American Type Culture Collection (ATCC, Rockville, MD, USA) supplied OCT2 (normal human proximal renal tubular epithelial cell line) and A498, 786-O, Caki-1, and ACHN (renal cancer cell lines). DMEM (Thermo Fisher HyClone, Logan, UT, USA) incorporating 10% FBS (Thermo Fisher Scientific, Waltham, MA, USA) was adopted as the culture medium. The cells were kept in an incubator (37° C, 5% CO2). With the medium refreshed every 2 days, the cells were passed once in 5 days. When the cells grew to about 90% of the bottle bottom, the overexpression plasmids of FSTL3, small interfering RNA targeting FSLT3 (si-FSTL3) or β-catenin (si-β-catenin), and their controls were transfected into A498 and ACHN cells using Lipofectamine3000 (Invitrogen, Carlsbad, CA, USA). Then the transiently transfected cells were screened and gathered for the following experiments. The β-catenin inhibitor XAV939 (MedChemExpress, Cat.No. HY-15147, 1 μM) was administered to restrain β-catenin in accordance with the producer’s instructions.

### Reverse transcription-quantitative PCR (RT-qPCR)

The total RNA in the A498 and ACHN cell lines was extracted with the application of the TRIzol reagent (Invitrogen, Carlsbad, CA, USA), and the mRNA was reverse-transcribed into cDNA with the RevertAid First Strand cDNA Synthesis kit (Thermo Fisher Scientific, Waltham, MA, USA). The LightCycler®480 system (Roche, Switzerland) and SYBR Green qPCR Master Mix (MedChemExpress, Monmouth Junction, NJ, USA) reagents were deployed for real-time fluorescent quantitative PCR. The 2^-ΔΔCt^ approach was taken to assess FSTL3 expression level (GAPDH as the internal parameter). Table 1 for each molecular primer.

**Table 1:** The sequences of each molecular primer.

FSTL3	Forward: 5’-CTGGGATCCTGAGCACGTAT-3’
	Reverse: 5’-GCCAGGGTCCAATGTTTCTA-3’
GAPDH	Forward: 5’-TGGTTGAGCACAGGGTACTT-3’
	Reverse: 5’-CCAAGGAGTAAGACCCCTGG-3’

### BrdU assay

The single-cell suspensions of A498 and ACHN cells were prepared and inoculated into a 96-well plate (1×10^4^ cells/well). Twenty-four hours after incubation, BrdU solution (30 μmol/L, Beyotime, Shanghai, China) was added into the cells for 8-hour incubation at 37° C. Next, the cells were fixed by 4% paraformaldehyde at indoor temperature for 30 min before they were flushed with PBS three times. 0.1% Tween-20 was utilized for permeation for 30 min at 37° C. After that, 5% BSA (Sigma-Aldrich) was applied to block the cells for 1 h at indoor temperature, which were then incubated along with the primary anti-BrdU antibody (1:500; cat. no. ab6326; Abcam) for 2 h at 37° C. After being rinsed three times with PBS, the cells were incubated with Alexa Fluor® 488 Rat monoclonal [BU1/75 (ICR1)] to BrdU (1:200, ab220074, Abcam) for 1 h at indoor temperature. DAPI staining solution (Beyotime, Shanghai, China) was harnessed to dye the nuclei at 37° C for 30 min. A fluorescence microscope (magnification, ×400; Olympus Corporation) was manipulated to observe the cells and capture their images. Cell proliferation rate = the number of BrdU staining positive cells/total DAPI staining positive cells ×100%.

### CCK8 assay

The transiently transfected A498 and ACHN cells were prepared into single-cell suspension, which was inoculated in 96-well plates (2×10^3^ cells/well). After 24 h, 48 h, 72 h, and 96 h post seeding, 10 μl CCK8 solution (Beyotime Biotechnology, Wuhan, China) was administered into each hole for another one-hour incubation. The optical density (OD) was examined at 450 nm with the use of a Microplate reader.

### Flow cytometry

The transiently transfected A498 and ACHN cells were transformed into single-cell suspension, which was inoculated in 6-well plates (2×10^6^ cells/well). Cisplatin (5 μM) was adopted to treat A498 and ACHN cells for 24 hours to trigger apoptosis. With the density adjusted to 2×10^6^ cells/well, the cells were cultivated for another 24 hours, and the supernatant was then abandoned. After they were rinsed twice with pre-cooled PBS, cell apoptosis was examined strictly in conformity with the instructions of the apoptosis kit (BD556547; BD Biosciences, Franklin Lakes, NJ, USA). A Muse cell analyzer (Millipore, Darmstadt, Germany) was deployed to analyze apoptotic cell quantification.

### Transwell invasion experiment

The transfected cells were seeded onto the upper layer of a Transwell chamber (Millipore, Billerica, MA, USA) coated with Matrigel (BD Biosciences, San Jose, CA, USA). DMEM (200 μL) incorporating 20% FBS was put in the lower compartment as a chemo-attractant. As the cells were cultivated for 24 h, all uninvaded cells were cleared. Matrigel was fixed with paraformaldehyde, and then the crystal violet solution was adopted to dye the cells. A phase-contrast microscope (Olympus, Tokyo, Japan) was introduced to calculate the invaded cells. The procedure was repeated three times, and the measurement was taken three times.

### Western blot

After the cells were treated by different factors, the original culture medium was abandoned, and the RIPA (containing 1% PMSF) lysis buffer was applied to digest the cells and separate the total protein. The total protein concentration was examined applying the BCA approach (Thermo Fisher Scientific, Inc., Rockford, IL, USA). The protein lysates were isolated on 10% SDS-PAGE gel and electrophoretically moved onto PVDF membranes (Millipore, Minneapolis, MN, USA). The membranes were blocked with 5% skim milk for 1 h at indoor temperature and incubated overnight along with primary antibodies Anti-E-cadherin (ab76055, 1:1000, Abcam, USA), Anti-Twist (ab175430, 1:1000, Abcam, USA), Anti-Slug (ab27568, 1:1000, Abcam, USA), Anti-GSK3 beta (phospho S9) (ab75814, 1:1000, Abcam, USA), Anti-GSK3 alpha (phospho S21) (ab28808, 1:1000, Abcam, USA), Anti-GSK3 beta (ab93926, 1:1000, Abcam, USA), Anti-beta Catenin (ab32572, 1:1000, Abcam, USA), Anti-BMP1/PCP (ab205394, 1:1000, Abcam, USA), Anti-Smad1 (phospho S206) (ab106093), Anti-Smad1 (ab339021:1000, Abcam, USA), Anti-Smad2 (phospho S467) (ab2808881:1000, Abcam, USA), Anti-Smad2 (ab408551:1000, Abcam, USA), Anti-Smad3 (phospho S423 + S425) (ab52903, 1:1000, Abcam, USA), Anti-Smad3 (ab40854,1:1000, Abcam, USA), Anti-FSTL3 (cat.no. LS-C166265, LifeSpan Biosciences), and Anti-GAPDH (ab181602, 1:1000, Abcam, USA) at 4° C. When the membranes were flushed twice in TBST, the cells were incubated for 1 h with the fluorescein-labeled secondary antibody Goat Anti-Rabbit IgG (1:3000, ab150077, Abcam, USA) at indoor temperature. Following 3 times washing, the ECL chromogen was applied for exposure, and a membrane scanner was employed for imaging.

### Enzyme linked immunosorbent assay (ELISA)

The FSTL3 ELISA kit (Cat.No. GR2021-03, Jymbio, Colorful Gene Biological Technology, China) was deployed for FSTL3 level measurement in the culture medium of RCC cells. The transiently transfected A498 and ACHN cells were prepared into single-cell suspension, which was seeded into 6-well plates (1×10^6^ cells/well). After 24-hour culture, the supernatant from the medium was collected, and the suspended cells were cleared through 20-minute centrifugation at 2000 rpm. ELISA was implemented, as instructed by the manufacturer. The optical density of each hole was confirmed at 450 nm with the use of a microplate reader (SpectraMax iD5, Molecular Devices company, Shanghai, China).

### Tumorigenesis in nude mice

Female BALB/c mice (6-8 weeks old) were bought from the Shandong Experimental Animal Center. A498 cells transfected along with FSTL3 overexpression plasmids were taken, with the cell concentration adjusted to 2×10^8^ ml^-1^. 0.1 ml of the cell suspension was transfused subcutaneously into the left forearm armpit of each nude animal, 15 mice in total. The mice were reared without pathogens, given adequate food and water, and their survival was monitored. On the 12th day, the tumor size of the nude mice was measured with a caliper (every 4 days), with the volume = long diameter × short diameter 2/2. During the fourth week, the mice were sacrificed, and the weight of tumors in the newly dead mice was examined. Zaozhuang Municipal Hospital authorized the guidelines for the Care and Use of Experimental Animals. The experiments were implemented strictly in keeping with the guidelines.

### Immunohistochemical staining

The formed tumor tissues or clinical samples obtained from RCC patients were routinely embedded in paraffin and consecutively sliced up into coronal sections (about 4 μM thick) for immunohistochemical staining. The sections were dehydrated with ethanol subsequent to conventional paraffin embedding. Antigen repair was done at 100° C, and 5%BSA blocking solution was added. The slices were cultivated overnight at 4° C, with the addition of diluted primary antibodies Anti-E-cadherin (ab76055, 1:200, Abcam, USA) and Anti-FSTL3 (cat.no. LS-C166265, 1:50, LifeSpan Biosciences), after which they were cultured with the HRP-labeled anti-rabbit IgG secondary antibody Goat Anti-Rabbit IgG (1:2000, ab6721, Abcam, USA) (1 h, 37° C). The reagents SABC and DAB were administered for color development. After being slightly re-stained with hematoxylin, the cells went through dehydration and fixation.

### Statistical analysis

Statistical analysis was conducted with the help of the GraphPad Prism 6 software (GraphPad Software, Inc., San diego, CA, USA). The outcomes were displayed as mean±SD (x±s). As one-way ANOVA was taken to compare multiple factors, an independent sample t-test was deployed for the comparison between two different groups, with *P<0.05* was regarded as statistically valuable.

### Ethics statement

Our study was approved by the Ethics Review Board of Zaozhuang Municipal Hospital.

### Data availability statement

The data sets used and analyzed during the current study are available from the corresponding author on reasonable request.
